# Closing Editorial: Research Progress on Chitosan Applications

**DOI:** 10.3390/polym16243527

**Published:** 2024-12-18

**Authors:** William M. Facchinatto, Sérgio Paulo Campana-Filho

**Affiliations:** 1 Aveiro Institute of Materials, CICECO, Department of Chemistry, University of Aveiro, St. Santiago, 3810-193 Aveiro, Portugal; 2 São Carlos Institute of Chemistry, University of São Paulo, IQSC/USP, Ave. Trabalhador São-Carlense, 400, São Carlos 13560-970, SP, Brazil

## 1. Introduction

Chitosan has attracted significant attention due to its versatile properties, which make it an ideal candidate for varied biomedical and industrial applications. This Special Issue of *Polymers* will present recent innovations and challenges in developing chitosan-based biomaterials, addressing applications in drug delivery, tissue engineering, antimicrobial treatments, food preservation, and environmental remediation. The 14 papers included in this Special Issue explore novel strategies to optimize the structural, physical–chemical, mechanical, and bioactive properties of chitosan composites, highlighting the material’s prospectives.

## 2. An Overview of the Published Articles

The articles in this Special Issue highlight chitosan’s potential use in targeted therapies, regenerative medicine, and sustainable practices. Tan et al. discuss the encapsulation of essential oils in chitosan nanoparticles used for breast cancer treatment, where nanoencapsulation significantly enhances bioavailability and therapeutic efficacy against breast cancer cells [1]. Complementing this therapeutic approach, Milano et al. report on freeze-dried chitosan-based implants containing thrombin and platelet-rich plasma, optimized for tissue regeneration applications, showing efficient solidification properties that are very favorable in orthopedic surgeries [2].

Hengtrakool et al. present findings on modified chitosan resin–glass ionomer cement for odontological applications, which facilitates the prolonged release of bioactive molecules, promoting cellular proliferation while ensuring minimal fluoride release [3]. García-García et al. explore the use of chitosan with nanostructured ZnO for strawberry preservation, where the composite coating maintains fruit quality while extending shelf life due to its antimicrobial properties [4].

Addressing non-enzymatic cell detachment methods, Huang et al. introduce responsive chitosan microcarriers utilizing host–guest interactions, which allow for controlled cell detachment [5]. This innovation is beneficial in cell expansion and bioreactor systems, particularly where non-invasive cell retrieval is critical. Camilo et al. study the role of chitosan in enhancing bond strength in odontological applications, focusing on the material’s role in reinforcing adhesion between dentin and fiberglass pillars [6].

Durán et al. highlight thermosensitive chitosan hydrogels for prolonged iron supplementation, providing a solution for controlled nutrient release, particularly beneficial in veterinary applications [7]. Ortega-Sánchez et al. contribute to the field of tissue engineering with chitosan-based hydrogels designed for chondrocyte culture, showing structural compatibility and bioactivity conducive to auricular cartilage repair [8].

Correa et al. examine the antifungal properties of chitinase and chitin platforms, which exhibit strong fungicidal effects against *Lasiodiplodia theobromae*, a phytopathogen [9]. Their findings have potential for agricultural biocontrol. Zlotnikov et al. investigate Förster resonance energy transfer (FRET) probes within chitosan micelles, optimizing drug-loading efficiency and structural integrity for drug delivery applications [10].

Khubiev et al. introduce Rhodamine B-infused chitosan films with luminescent and antibacterial properties, suitable for packaging and clinical environments where antimicrobial efficacy and visual detection are beneficial [11]. Ipinza-Concha et al. present a chitosan–riboflavin bioconjugate effective against green mold in citrus fruits, offering a natural alternative to synthetic fungicides [12].

Doan et al. further expand on chitosan’s versatility by detailing two studies [13,14]. The first one explores silver nanoparticle composites with chitosan, polyethylene glycol, polyvinyl alcohol, and polyvinylpyrrolidone, optimized as potent antibacterial agents against pathogens such as *Staphylococcus aureus*, *Pseudomonas aeruginosa*, and *Salmonella enterica*. This study underscores the material’s potential uses in antimicrobial applications [13]. The second study presents superparamagnetic iron oxide nanoparticles modified with chitosan and similar polymers, demonstrating their efficacy as methylene blue adsorbents in water treatment applications. This innovative approach highlights chitosan’s role in addressing environmental issues [14].

These 14 articles collectively illustrate the chitosan-based material’s adaptability, discussing potential modifications to enhance its mechanical and bioactive properties for diverse applications, ranging from environmental sustainability to medical therapies.

## 3. Conclusions

This Special Issue underscores the breadth of chitosan-based innovations and their potential to address real-world challenges. From advanced drug delivery systems to eco-friendly preservation solutions, the studies collectively highlight chitosan’s adaptability, biodegradability, and efficacy. As research advances, the further optimization of chitosan’s properties could lead to more targeted and efficient applications in both industrial and clinical settings.
